# Molecular and toxicogenetic safety profile of short-term nitrous oxide anesthesia in surgical patients

**DOI:** 10.1007/s11033-026-12264-1

**Published:** 2026-07-01

**Authors:** Juliana R. Lara, Flávia R. Nogueira, C.-Y. Oliver Chen, Mariane A. P. Silva, José Reinaldo C. Braz, Leandro G. Braz, Mariana G. Braz

**Affiliations:** 1 São Paulo State University (UNESP), Medical School, Division of Anesthesiology, GENOTOX Lab, Botucatu, Brazil; 2 Biofortis Research, Merieux NutriSciences, Addison, IL USA; 3 UNESP - Botucatu Medical School, Prof. Mario Rubens G. Montenegro Av., Botucatu, Sao Paulo, 18618-687 Brazil

**Keywords:** Nitrous oxide, DNA damage, Gene expression, Homocysteine, B vitamins, Antioxidant

## Abstract

**Background:**

Nitrous oxide (N2O) is a widely used anesthetic; however, its potential to compromise genomic stability by disrupting vitamin B12 and folate metabolism remains a critical concern in clinical molecular biology. Since N2O can oxidize cobalamin, it may theoretically impair the methionine synthase pathway, leading to DNA damage. Thus, this study aimed to evaluate the toxicogenetic impact—DNA damage and gene expression—and the metabolic profile of vitamins B9 and B12, homocysteine (HCY) and micronutrients/antioxidants in patients undergoing short-term N2O anesthesia.

**Methods and Results:**

Adult surgical patients without comorbidities (*n* = 18/group) were assessed at: pre-anesthesia, during anesthesia (1.5 h exposure), and 24 h post-anesthesia. Both groups received desflurane, with one also receiving N2O. The primary focus was on DNA strand breaks (comet assay) and the transcriptional levels (RT-qPCR) of repair (*OGG1* and *XRCC1*) and antioxidant (*HO-1*) genes, followed by HPLC and chemiluminescence analysis of metabolic markers (HCY and antioxidants, vitamins B9 and B12). N2O exposure did not modulate the mRNA expression of any of the studied genes, nor did it alter HCY, vitamin levels or antioxidants (*p* > 0.05). A transient, group-independent increase in DNA damage was observed post-anesthesia, suggesting a primary response to surgical stress rather than the anesthetic agent.

**Conclusions:**

Our study provides high-evidence molecular data demonstrating that 1.5 h of N2O exposure is safe for genomic integrity and vitamin B-related metabolism in healthy individuals. These findings are clinically relevant as they validate the continued use of N2O in minimally invasive procedures without the risk of acute toxicogenetic or metabolic impairment.

## Introduction

Maintaining genomic stability is essential for cellular homeostasis, yet many pharmacological agents used in clinical practice, such as anesthetics, have poorly understood molecular side effects. Nitrous oxide (N2O), an anesthetic gas, may disrupt key biosynthetic pathway [1]. The molecular concern regarding N2O is its ability to oxidize the cobalt atom of vitamin B12 (cobalamin), irreversibly inhibiting methionine synthase. This enzyme is a key regulator of the folate cycle, mediating the transfer of methyl groups from methyltetrahydrofolate to homocysteine (HCY) to generate methionine and S-adenosylmethionine—essential precursors for DNA, RNA, and myelin synthesis [2].

Disruption of this metabolic axis can lead to hyperhomocysteinemia, which is associated with oxidative stress, endothelial dysfunction, and increased cardiovascular risk [2, 3]. From a toxicogenetic perspective, impaired folate metabolism and increased HCY are linked to DNA strand-breaks and reduced DNA repair capacity. While some studies [4–8] have explored vitamin B and HCY fluctuations in patients with pre-existing comorbidities or undergoing invasive/major surgeries there is a critical gap in the literature regarding the impact of N2O on genomic integrity and gene expression in healthy individuals. Specifically, the modulation of key DNA repair genes, such as 8-oxoguanine DNA glycosylase 1 (*OGG1*) and X-ray repair cross-complementing group 1 (*XRCC*1), and antioxidant response like heme oxygenase-1 (*HO − 1*), remains largely unexplored in the context of anesthesia with N2O.

Inhalational agents like desflurane are often preferred for their low solubility and rapid elimination, yet their molecular interaction with N2O in short-term procedures has not been fully characterized [9]. Given the conflicting findings related to the modulation of gene expression by anesthetic agents other than N2O in blood cells [10–12] it is vital to determine whether N2O poses a modulation of transcripts in minimally invasive surgeries.

This study aimed to provide a comprehensive molecular and toxicogenetic evaluation of N2O anesthesia in healthy surgical patients. For the first time, we integrated an analysis of DNA damage and transcriptional regulation with the assessment of the folate cycle and redox status. Our findings offer novel insights into the safety profile of N2O, establishing whether short-term exposure triggers acute molecular impairment in a surgical population.

## Materials and methods

### Patients and anesthesia procedure

After UNESP approval of this study (#91391518.3.0000.5411), which was carried out in accordance with the Declaration of Helsinki, all of the participants answered a questionnaire about their lifestyle, health status and previous exposure to environmental pollutants and signed an informed consent form. The inclusion criteria included patients classified as physical status I (healthy patients with no disease other than a surgical abnormality) who were scheduled for elective minimally invasive surgery (septoplasty) lasting 1.5 h. The exclusion criteria included patients younger than 18 years, elderly patients, smokers, alcohol consumers, obese patients, pregnant women, patients with recent consumption of antioxidant supplements, patients with radiation exposure/treatment and patients who were undergoing other anesthesia procedures to avoid possible bias.

Briefly, all of the patients (*n* = 36, 18 patients/group) were monitored (temperature, hemodynamic and respiratory parameters) under general anesthesia with desflurane. All patients received active warming to maintain normothermia. The same protocol was used for both groups of patients, except that one group received N2O during the maintenance of anesthesia and the other group did not. Patients were premedicated and after preoxygenation (O_2_), anesthesia induction was performed with fentanyl and propofol; rocuronium bromide was administered to facilitate orotracheal intubation and maintain adequate neuromuscular blockade during surgery. The flow rate (2 l/min) was used in both groups, with a mixture of O_2_ (40%) and air in the group without N_2_O and with a mixture of O_2_ (40%) and N2O (60%) in the N_2_O group. Postoperative analgesia and prophylaxis against nausea and vomiting were administered to all the patients at the end of the surgery. After extubation, the patients were immediately transferred to the postanesthesia care unit, where none of them experienced complications.

### Blood sampling and time point analysis

Venous blood samples were collected from fasted patients in both groups: pre-anesthesia, during anesthesia (1.5 h exposure) and 24 h post-anesthesia. The samples were drawn in tubes with the anticoagulant ethylenediaminetetraacetic acid (EDTA), with separating gel and PAXgene Blood RNA tubes^®^. The coded samples were protected from light and immediately processed in the laboratory; all the experiments were run in duplicate and analyzed blindly. Some of the samples were frozen in small aliquots in a -80 °C freezer until analysis of the proposed markers. Quality plasma or serum control samples were used in the assays. The experiments were run under red or yellow light (comet assay).

### Evaluation of DNA damage

Peripheral blood mononuclear cells (PBMCs) were used for the comet assay, which was conducted according to a protocol [10] following established guidelines [13]. Briefly, PBMCs were mixed with 0.5% low-melting-point agarose at 37 °C. The mixture was pipetted onto coded microscope slides previously covered with a layer of 1.5% normal-melting-point-agarose, covered with coverslips and left at 4 °C to solidify; then, the coverslips were carefully discarded. Next, the slides were immersed in fresh cold lysis solution overnight. The slides were then washed in saline solution and added to a horizontal electrophoresis tank (immersed on ice) with cold fresh buffer (1 mM EDTA and 300 mM NaOH, pH > 13) for 40 min. The slides were subsequently subjected to 30 -min of electrophoresis at 0.8 V/cm and 300 mA. Each electrophoretic run was checked against positive and negative controls. Following neutralization (0.4 M Tris-HCl, pH 7.5), the slides were fixed with ethanol and air-dried. The slides were then stained with SYBR Gold and immediately analyzed under fluorescence connected to a Comet Assay IV image analysis system (Perceptive Instruments, UK); images from 100 nucleoids were scored from each time point/patient. The % DNA in the tail was the parameter used to estimate DNA damage.

### Evaluation of relative gene expression

After the tubes were thawed, RNA from all the patients (time points) was extracted according to the manufacturer’s protocol (PAXgene kit, Qiagen/PreAnalytics^®^). The RNA quality was determined by spectrophotometry, which revealed that the A260/280 ratio ranged from 1.9 to 2.0. Integrity was analyzed via a 2100 Bionalyzer device, and RNA integrity number values were equal to or greater than 8, which is considered ideal for determining gene expression.

The RNA samples were then transcribed into complementary DNA (cDNA) via a reverse transcription kit according to the manufacturer’s protocol (Applied Biosystems - ABI, USA), with the following cycling steps: 25 °C for 10 min, incubation for 120 min at 37 °C, and then incubation at 4 °C. The cDNA samples were stored at -80 °C until transcript detection. Relative quantification of transcripts was performed in a real-time automatic thermocycler (ABI 7500 Fast, USA) via TaqMan assays (Thermo Fisher Scientific, USA) for *XRCC1* (Hs00959834_m1), *OGG1* (Hs00213454_m1), and *HO-1* (Hs01110250_m1). The selected endogenous control (housekeeping) was the ß-actin gene - *ACTB* (Hs99999903_m1).

A pool of cDNA samples from healthy subjects who did not undergo surgery was used as a control to determine the relative quantification in each 96-well plate, in accordance with previous protocols [11, 14], and a negative control was added to ensure that no contamination was present. To prepare each plate, 300 µl of Fast Universal PCR Master Mix, 150 µl of DNase/RNase-free water and 30 µl of probe were added in a final volume of 10 µl (8 µl of the mixture and 2 µl of cDNA), with the following thermocycling conditions for all genes: 94 °C for 10 min, followed by 40 cycles at 95 °C for 15 s and 60 °C for 60 s. The 2^−ΔΔCt^ formula was applied for relative quantification of gene expression [15].

### Detection of folate, cobalamin and homocysteine

Serum vitamins B9 and B12 were detected by chemiluminescence via IMMULITE 2000 kits (Siemens Healthcare Diagnostics, USA). The assessment of plasma HCY was carried out via high-performance liquid chromatography- HPLC with a fluorescence detector (Perkin Elmer 650 − 15 Fluorescence Spectrophotometer, USA). The reference values were 3–17 ng/ml for B9, 193–982 pg/ml for B12, and 6–15 µM for HCY.

### Assessment of systemic lipophilic antioxidants

Using HPLC, the fat-soluble antioxidants carotenoids and tocopherols were evaluated according to a previously described protocol [16], with some modifications. Serum samples were extracted in lipophilic solvents (chloroform/methanol − 2:1) and centrifuged. Next, hexane was added, and the samples were subjected to another round of centrifugation. Echinenone was added as an internal standard, and the supernatant was filtered through a nylon membrane and dried in nitrogen gas. After, the samples were resuspended in HPLC-grade ethanol and then injected into a Waters Alliance 2695 separation module chromatograph, and the components were separated via a C30 column (3 μm, 150 × 3.0 mm, YMC, USA).

The mobile phases were composed of solution A containing ammonium acetate in Milli-Q^®^ water: methanol: methyl tert-butyl ether- MBTE and solution B containing ammonium acetate in Milli-Q^®^ water: methanol: MBTE via gradient elution at a flow rate of 1 ml/min over 32 min. The antioxidants were identified by a photodiode detector (Waters PDA 2998 detector, USA) adjusted to 450 nm for the evaluation of carotenoids (α- and β-carotene) and 292 nm for tocopherols (α- and γ-tocopherol). The results were adjusted with an internal standard (control), which was injected daily during the execution of the experiments. The data were analyzed by using software coupled with equipment to obtain individual dosages. Each compound was identified and quantified according to the area of the peaks presented in the chromatograms.

### Statistical analysis

The sample size was calculated assuming a difference of 2.1% between the % DNA in the tail mean values of the groups and a standard deviation of 2.0%. A minimum of 18 patients/group was necessary to detect this difference via a 2-tailed test, with a type I error probability of 0.05 and a type II error of 0.12 (test power: 88%).

The parameters were compared between the two groups at different time points via profile analysis (repeated-measures analysis of variance). In this analysis, the following hypotheses were tested: there would be significant differences between mean groups over time, and there would be an interaction between groups and time points. This analysis was followed by 95% confidence intervals (CIs) with Bonferroni correction for multiple comparisons of time points in each group. The significance level was set at *p* < 0.05.

## Results

The study groups were homogeneous regarding demographic characteristics and intraoperative parameters (*p* > 0.05, data not shown), ensuring a balanced comparison for toxicogenetic and metabolic endpoints.

Regarding DNA damage (Table 1), no significant inter-group differences or time-group interactions were observed. However, a significant time-point effect was identified (*p* = 0.004); the overall mean of DNA strand breaks increased 24 h post-anesthesia in both groups, regardless of N2O administration. In contrast, the mRNA expression of DNA repair (*XRCC1* and *OGG1*) and antioxidant (*HO-1*) genes showed no significant fluctuations. There were no differences between time points or groups, indicating that transcriptional regulation of these pathways was not acutely modulated by the anesthetic protocol (Table 2).

**Table 1:** DNA damage (% DNA in the tail) evaluated in peripheral blood mononuclear cells from anesthetized patients

Groups	Time points			Mean groups over time
	Before	During	After	
- N_2_O	7.0 ± 4.9 (4.0; 10.0)	8.2 ± 5.4 (5.0; 11.5)	10.1 ± 5.7^*^ (5.7; 12.6)	8.1 (5.3) (6.3; 10.0)
+ N_2_O	8.8 ± 6.1 (5.0; 11.2)	9.3 ± 6.2 (5.6; 12.9)	12.2 ± 7.4^*^ (7.1; 17.3)	10.4 (7.2) (7.9; 13.0)

With (+) or without (-) N2O = nitrous oxide. Data are shown as the mean ± standard deviation (95% confidence interval using Bonferroni correction for three time points) and mean groups over time (standard deviation) [95% confidence interval]. P values > 0.05 for difference between mean groups over time and for interaction effects from repeated-measures analysis collapsing over time. The general mean was greater at the last time point regardless of the group (after anesthesia; ^*^P = 0.004)

**Table 2:** Relative expression of genes *XRCC1*,* OGG1* and *HO-1* detected in leukocytes from anesthetized patients

Groups	Gene	Time points			Mean groups over time
		Before	During	After	
- N_2_O	*XRCC1*	1.07 ± 0.19 (0.79; 1.26)	1.12 ± 0.21 (0.78; 1.55)	0.82 ± 0.12 (0.57; 1.07)	1.00 (0.15) (0.70; 1.30)
+ N_2_O		0.90 ± 0.21 (0.84; 1.19)	1.06 ± 0.24 (0.87; 1.19)	0.75 ± 0.13 (0.91; 1.32)	0.94 (0.17) (0.71; 1.17)
- N_2_O	*OGG1*	1.12 ± 0.09 (1.12; 1.28)	1.17 ± 0.08 (1.00; 1.33)	1.20 ± 0.10 (1.02; 1.45)	1.13 (0.06) (1.11; 1.35)
+ N_2_O		1.02 ± 0.09 (0.94; 1.19)	1.07 ± 0.09 (0.97; 1.29)	1.11 ± 0.10 (0.91; 1.32)	1.05 (0.05) (0.94; 1.17)
- N_2_O	*HO-1*	1.19 ± 0.39 (0.98; 1,39)	1.22 ± 0.52 (0.84; 1.31)	1.30 ± 0.57 (0.83; 1.78)	1.24 (0.47) (0.85; 2.00)
+ N_2_O		1.16 ± 0.40 (0.92; 1,30)	1.19 ± 0.55 (0.95; 1.42)	1.28 ± 0.59 (0.85; 1.91)	1.27 (0.49) (0.81; 1.57)

With (+) or without (-) N_2_O = nitrous oxide. Data are shown as the mean ± standard deviation (95% confidence interval using Bonferroni correction for three time points) and mean groups over time (standard error of the mean) [95% confidence interval]. P values > 0.05 for difference between mean groups over time and for interaction effects from repeated-measures analysis collapsing over time

Vitamin B9, B12, and HCY levels remained within clinical reference limits across all assessments (Table 3). No significant alterations were observed over time or between groups (*p* > 0.05). Similarly, systemic lipophilic antioxidant levels (carotenes and tocopherols) remained stable (Table 4). No significant differences were found regarding mean concentrations, time-point variations, or group interactions, suggesting that redox homeostasis was preserved during and after short-term N2O exposure.

**Table 3:** Detection of cobalamin (vitamin B12), folate (B9) and homocysteine (HCY) in both groups of anesthetized patients

Groups	Markers	Time points			Mean groups over time
		Before	During	After	
- N_2_O	B12 (pg/ml)	416.0 ± 37.9 (339.2; 492.8)	354.6 ± 33.1 (287.5; 421.6)	377.3 ± 33.6 (309.4; 445.3)	382.6 (34.3) (313.1; 452.1)
+ N_2_O		422.0 ± 37.9 (345.2; 498.8)	355.2 ± 33.1 (288.1; 422.2)	372.2 ± 33.6 (304.2; 440.1)	383.1 (34.3) (313.7; 452.6)
- N_2_O	B9 (ng/ml)	13.3 ± 1.4 (10.5; 16.2)	11.9 ± 1.4 (9.1; 14.7)	10.6 ± 1.4 (7.7; 13.4)	11.9 (1.3) (9.2; 14.6)
+ N_2_O		14.1 ± 1.4 (11.3; 16.9)	13.1 ± 1.3 (10.4; 15.8)	11.7 ± 1.4 (8.9; 14.5)	13.0 (1.3) (10.3; 15.6)
- N_2_O	HCY (µM)	8.4 ± 0.8 (6.8; 10.1)	8.2 ± 1.1 (6.1; 10.4)	5.5 ± 0.8 (4.0; 7.1)	7.4 (0.8) (5.7; 9.1)
+ N_2_O		9.1 ± 0.8 (7.4; 10.7)	9.0 ± 1.1 (6.9; 11.2)	7.1 ± 0.8 (5.5; 8.6)	8.4 (0.8) (6.7; 10.1)

With (+) or without (-) N_2_O = nitrous oxide. Data are shown as the mean ± standard deviation (95% confidence interval using Bonferroni correction for three time points) and mean groups over time (standard error of mean) [95% confidence interval]. P values > 0.05 for difference between mean groups over time and for interaction effects from repeated-measures analysis collapsing over time

**Table 4:** Assessment of lipophilic antioxidants in both studied groups of anesthetized patients

Groups	Antioxidant	Time points			Mean groups over time
		Before	During	After	
- N_2_O	α-carotene (µM)	0.21 ± 0.03 (0.15; 0.26)	0.22 ± 0.03 (0.17; 0.28)	0.27 ± 0.04 (0.20; 0.35)	0.23 (0.02) (0.18; 0.28)
+ N_2_O		0.25 ± 0.02 (0.20; 0.30)	0.24 ± 0.02 (0.19; 0.29)	0.28 ± 0.03 (0.20; 0.35)	0.26 (0.02) (0.21; 0.30)
- N_2_O	β-carotene (µM)	0.31 ± 0.05 (0.20; 0.42)	0.27 ± 0.05 (0.17; 0.36)	0.33 ± 0.05 (0.22; 0.44)	0.30 (0.04) (0.21; 0.39)
+ N_2_O		0.34 ± 0.05 (0.24; 0.44)	0.35 ± 0.04 (0.26; 0.43)	0.30 ± 0.05 (0.20; 0.40)	0.33 (0.04) (0.25; 0.41)
- N_2_O	α-tocopherol (µM)	26.9 ± 1.3 (24.3; 29.6)	24.4 ± 1.4 (21.7; 27.2)	24.0 ± 1.3 (21.4; 26.6)	25.1 (1.3) (22.5; 27.8)
+ N_2_O		24.2 ± 1.2 (21.7; 26.7)	22.5 ± 1.3 (19.9; 25.1)	22.8 ± 1.2 (20.3; 25.2)	23.1 (1.2) (20.6; 25.6)
- N_2_O	γ-tocopherol (µM)	3.7 ± 0.3 (3.2; 4.3)	3.3 ± 0.3 (2.8; 3.9)	3.3 ± 0.3 (2.7–3.9)	3.5 (0.3) (2.9; 4.0)
+ N_2_O		3.9 ± 0.2 (3.4; 4.4)	3.6 ± 0.2 (3.1; 4.1)	3.6 ± 0.2 (3.1; 4.1)	3.7 (0.2) (3.2; 4.2)

With (+) or without (-) N_2_O = nitrous oxide. Data are shown as the mean ± standard deviation (95% confidence interval using Bonferroni correction for three time points) and mean groups over time (standard error of mean) [95% confidence interval]. P values > 0.05 for difference between mean groups over time and for interaction effects from repeated-measures analysis collapsing over time

## Discussion

A significant increase in DNA damage in PBMCs 24 h post-anesthesia, regardless of the use of N2O, was reported. This observation aligns with previous studies in patients undergoing minor surgery under desflurane, which also reported increased DNA strand-breaks postoperatively [17, 18]. Crucially, our data confirm that N2O does not exacerbate DNA damage beyond the effects of the surgical procedure itself [19]. Interestingly, although DNA damage was detected in PBMCs, we observed no transcriptional modulation of the repair genes *XRCC1* and *OGG1* or the antioxidant marker *HO-1*. This lack of response in peripheral blood leukocytes suggests that the transient genomic instability observed was likely a localized or low-magnitude effect of systemic surgical stress, insufficient to trigger a robust compensatory change in the expression of these specific DNA repair pathways. The stability of *HO-1* expression is further corroborated by the preserved systemic antioxidant levels observed in our study.

Regarding the metabolic axis, this study reveals that healthy patients undergoing minimally invasive surgery (1.5 h) do not present relevant changes in vitamins B9 and B12 or HCY levels. These results are particularly relevant as less invasive procedures typically result in reduced surgical trauma, lower inflammatory responses, and less oxidative stress compared to major surgeries [20]. Therefore, in this specific clinical context, 60% N2O exposure did not compromise folate cycle homeostasis.

In contrast to our findings, previous report [8] identified increased plasma HCY and vitamin B9 in patients with comorbidities undergoing major, long-duration invasive surgeries under N2O (combined with isoflurane, sevoflurane, or desflurane). Those authors noted that longer anesthesia times—and consequently higher cumulative N2O doses—correlated with higher HCY concentrations, suggesting that elderly, vegetarian/vegan, or folate-deficient individuals may face increased cardiovascular risk due to post-exposure hyperhomocysteinemia. Similarly, studies in pediatric patients [21] and adults undergoing anesthesia longer than 3 h [7] found postoperative HCY elevations and decreased vitamin B12 levels. Mechanistically, prolonged N2O exposure is known to oxidize the cobalt atom of vitamin B12, irreversibly inhibiting methionine synthase and interfering with nucleic acid and protein synthesis [22].

However, our results are consistent with research in patients over 55 years undergoing ophthalmological surgery [6], which showed that short-term N2O anesthesia (40–80 min) did not affect B12 levels. As suggested by a previous study [9], the clinical inhibition of methionine synthase may require a threshold of prolonged exposure. In healthy individuals undergoing short procedures (< 2 h), N2O appears to be safe regarding the folate and vitamin B12 pathways, with all markers remaining within physiological limits.

Finally, we observed that patients anesthetized with desflurane, with or without N2O, showed no impairment of systemic lipophilic antioxidants (tocopherols and carotenoids). This is consistent with studies reporting no differences in systemic oxidative stress or inflammatory profiles [18, 19]. While some literature describes increased antioxidant consumption 6 h after laparoscopic surgery—particularly when N2O was combined with desflurane [23]—that study focused only on the postoperative period. To our knowledge, this is the first study to demonstrate that redox homeostasis, evaluated via lipophilic antioxidants, remains preserved both intraoperatively and postoperatively during short-term N2O/desflurane anesthesia in healthy patients.

A potential limitation of this study is the sample size of 18 patients/group, which may limit the generalizability of our findings. However, it is important to emphasize that an a priori sample size calculation was performed, ensuring a robust statistical power of nearly 90% to detect significant differences.

## Conclusion

Our study demonstrates that short-term (1.5 h) administration of 60% N2O in healthy surgical patients does not compromise genomic integrity or transcriptional regulation of repair pathways. While a transient, group-independent increase in DNA damage was observed 24 h post-anesthesia—reflecting a general response to systemic surgical stress—N2O exposure did not exacerbate DNA strand-breaks or modulate the mRNA expression of DNA repair and antioxidant genes. Furthermore, this anesthetic protocol did not disrupt the metabolic homeostasis of the folate cycle, as evidenced by stable levels of vitamins B9, B12, and HCY, nor did it deplete systemic lipophilic antioxidants. These findings provide high-evidence molecular data supporting the toxicogenetic and metabolic safety of N2O in minimally invasive procedures. By establishing these safety parameters in a healthy population, our study offers relevant insights into clinical toxicology and strengthens the evidence base for safe anesthetic practice in modern surgery.

## Data Availability

The data of this study are available from the corresponding author upon request.
